# Effects on health and process outcomes of physiotherapist-led orthopaedic triage for patients with musculoskeletal disorders: a systematic review of comparative studies

**DOI:** 10.1186/s12891-020-03673-9

**Published:** 2020-10-10

**Authors:** K. S. Samsson, K. Grimmer, M. E. H. Larsson, J. Morris, S. Bernhardsson

**Affiliations:** 1 Region Västra Götaland, Research and Development Primary Health Care, Gothenburg, Sweden; 2 Department of Health and Rehabilitation, Institute of Neuroscience and Physiology, Sahlgrenska Academy, University of Gothenburg, Gothenburg, Sweden; 3 GHP Ortho Center Gothenburg, Gothenburg, Sweden; 4 Department of Physiotherapy, Stellenbosch University, Cape Town, South Africa; 5 University of South Australia, Adelaide, Australia; 6 The Canberra Hospital, Canberra, Australia

**Keywords:** Orthopaedic, Triage, Advanced practice physiotherapist, Extended scope physiotherapist, Patient-reported outcomes, Surgery conversion rate

## Abstract

**Background:**

Physiotherapist-led (PT-led) orthopaedic triage is an evolving model of care for patients with musculoskeletal disorders. Objectives for this study were to establish the current evidence body on the impact of PT-led orthopaedic triage on health, quality, and service outcomes for patients referred for orthopaedic consultation, compared with standard (orthopaedic surgeon) care.

**Methods:**

Medline, EMBASE, Scopus and CINAHL were searched from inception until 7 May 2018; search updated 24 April 2020. Search terms (including derivatives) included physiotherapy, advanced/extended scope, musculoskeletal/orthopaedic, triage. The search was framed as *Population* ***=*** patients referred for orthopaedic consultation; *Intervention* = PT-led orthopaedic triage; *Comparison* = standard care; *Outcomes* = health, quality and process outcomes. Only randomised controlled trials (RCTs) and prospective comparative cohort studies were eligible for inclusion. Screening, study selection, data extraction, and assessment of methodological quality were performed independently by reviewer pairs. Quality was scored with the Downs and Black checklist. Certainty of evidence was determined using GRADE. PROSPERO registration number CRD42017070950.

**Results:**

We included two RCTs and eleven cohort studies (*n* = 1357 participants) of variable methodological quality (range 14–23 of possible 28). Certainty of evidence was low to moderate. There was no difference between PT-led orthopaedic triage and standard care for patient-reported outcomes (two RCTs). Perceived quality of care with PT-led orthopaedic triage was higher (two RCTs, four cohort studies) or equal (one cohort study) compared with standard care. PT-led orthopaedic triage had higher surgery conversion rates (one RCT, three cohort studies) (55–91% vs 22–38%), lower (two RCTs) or equal rate (two cohort studies) of referral for investigations, shorter waiting times (one RCT, one cohort study), and lower costs (one RCT). Furthermore, there was high agreement between physiotherapists’ and orthopaedic surgeons’ treatment approach (eight cohort studies), referral for investigation (five cohort studies), and diagnosis (nine cohort studies). Study limitations were **t**he low number of RCTs, and variable methodological quality.

**Conclusions:**

Evidence of low to moderate certainty suggests that PT-led orthopaedic triage leads to similar diagnostic decisions as standard care, has a higher conversion-to-surgery rate, reduces waiting times, is cost effective and valued by patients, and that health outcomes are equivalent.

## Background

According to Global Burden of Disease studies, musculoskeletal disorders have been the leading cause of disability for 30 years [1–5], and, due to population aging, are expected to rise significantly over the coming decades [6]. Patients with musculoskeletal disorders comprise up to 30% of consultations in primary care [7–11], making this the second-highest reason for consulting a general medical practitioner (GP) [10]. In most countries, it is standard practice for GPs to refer to a hospital-based orthopaedic surgeon (OS) for advice, investigations, and/or interventions, [12–14] although it is believed that fewer than 40% of patients [12, 15–17] require an orthopaedic intervention. Thus the urgent challenge facing current health services internationally is to improve access to timely, high quality orthopaedic consultations for the people who need them most, whilst offering equally effective and cost effective alternative care pathways for people who may not require orthopaedic surgeon intervention [18].

Consequently, during the latest two decades, alternative models of care, such as physiotherapist-led (PT-led) orthopaedic triage, have been explored, predominantly in Australia [19], Canada [20, 21], Ireland [22], UK [23] and Sweden [24]. This model of care involves a physiotherapist (PT) assessing, diagnosing and managing patients referred for orthopaedic consultation; a procedure normally done by an OS [25, 26]. This model could potentially be cost effective. Morris et al. [27] reported that appointments with a PT are significantly less expensive than appointments with an OS, and data from four countries suggest that OSs cost the health system approximately twice as much as advanced practice PTs [28–36].

When implementing alternative models of care, evaluating effects on patients’ health outcomes is essential. Musculoskeletal disorders burden the individual, the family and society through pain, and disability [37], often limiting participation in daily life activities [37–39] and negatively influencing health-related quality of life [40–42]. Additionally, patients’ perceptions should be considered, particularly when interventions differ from traditional scope of practice [43, 44]. Patients’ reports of their experiences are increasingly recognised as a valid measure of quality in health care, as well as clinical effectiveness and safety [45, 46].

Several systematic reviews have been published comparing outcomes of advanced or extended scope of PT-led orthopaedic triage with standard care [47–50], however, these reviews differ in scope, comparators, and outcomes, include studies on specific populations, and were published in 2014 and 2015. Furthermore, the reviews concluded that the generally poor methodological quality of included studies, and low certainty of evidence, did not provide evidence for benefits of advanced or extended scope PT-led orthopaedic triage.

The fields of advanced and extended scopes of are continually developing, underpinned by an expanding body of evidence. An up-to-date review was therefore required to collate the current body of evidence for PT-led orthopaedic triage for musculoskeletal conditions on health and process outcomes. Objectives for this study were to establish the current evidence body on the impact of PT-led orthopaedic triage on health, quality, and service outcomes for patients referred for orthopaedic consultation, compared with standard (orthopaedic surgeon) care.

## Methods

### Protocol registration

The review protocol was registered on PROSPERO on 30-06-2017 (CRD42017070950). The protocol was modified to include prospective observational studies during the review process, due to the paucity of randomised controlled trials (RCTs) identified in the searches.

### Quality framework and reporting standard

The review is reported according to the Preferred Reporting Items for Systematic Reviews and Meta-Analyses (PRISMA) statement [51], and was undertaken in accordance with Cochrane guidelines [52].

### Search strategy

The electronic library databases Medline, EMBASE, Scopus and CINAHL were searched from inception to 7 May 2018. An updated search was performed on 24 April 2020. Reference lists of studies meeting the eligibility criteria were screened for additional relevant studies. Furthermore, a search in the database ClinicalTrials.gov yielded 34 hits, none of which were relevant for our review. A comprehensive search strategy was developed with support from a medical librarian, using a combination of keywords and MeSH/thesaurus terms. An example of the search terms is outlined in Table 1. No limitations were placed on language or publication status.

**Table 1:** Search terms

#1	Physical therap*/physiotherapy* AND advance*/specialist*/experience*/expand*/extend*/scope of practice OR APP/ESP
#2	Musculoskeletal OR orthopaedic*/orthopedic*
#3	Triag*/assess*/screen*/manag*/diagnos*
#4	Exclude Emergency/trauma OR pediatric*/child*

### Eligibility criteria

Eligibility criteria were defined using a PICOS model (Population, Intervention, Comparator, Outcome and Study design) [53–55] reported in Table 2.

**Table 2:** PICOS for the study

Population	- Physiotherapists working in advanced or extended scope of practice, or in a role previously carried out by a member of the medical profession - Adults with orthopaedic/musculoskeletal disorders referred for orthopaedic consultation in all healthcare settings except emergency care
Intervention	- Orthopaedic/musculoskeletal triage led by physiotherapists, i.e. substitution of a physician with a physiotherapist
Comparison	- Standard care, i.e. referral by general practitioner and assessment by an orthopaedic surgeon
Outcomes	Primary outcomes - Patient-reported outcomes, including pain, disability, health state, psychological status, and health-related quality of life - Patient-reported experiences, including patients’ views of the quality of care and satisfaction with care received - Sick-leave Secondary outcomes - Process outcomes; surgery conversion rate (rate of patients that have gone on to have surgery), agreement on treatment approach (both clinicians agreeing regarding the patients’ need for conservative or surgical treatment approach), referral for investigation (the proportion of patients referred onward for investigations), agreement on referral for investigation (both clinicians agreeing that the patient needs investigation), agreement on diagnosis (both clinicians agreeing on the patient’s diagnosis.) - Waiting times - Cost effectiveness (direct or indirect costs)
Study design	- RCTs - Prospective decision-making agreement studies (inter-rater reliability studies) - Prospective, comparative studies (non-randomised experimental trials, controlled cohort studies, case-control studies and interrupted time series)
Setting	All healthcare settings except emergency care/trauma or pediatrics

### Exclusion criteria

Studies were excluded if triage was conducted by health professionals other than PTs; or the PTs conducting the triage were working in advanced or extended scope in specialties outside musculoskeletal / orthopaedics.

### Study selection

Two reviewers (KS and SB) independently screened titles and abstracts for eligibility using the online screening tool Rayyan [56]. Discrepancies were resolved by discussion. The full texts of potentially relevant papers were then retrieved and independently reviewed for eligibility, and differences were discussed when necessary. A third reviewer (ML) was consulted when the two reviewers could not reach consensus.

### Data extraction and analysis

Data extraction from included papers was performed by one reviewer (KS) and checked for accuracy by another reviewer (SB). Disagreements were resolved by discussion. Information was extracted on study characteristics (e.g. publication year, country, setting); patient characteristics (e.g. number of participants, age, gender); description of the triage model and any control interventions; follow-up; types of outcomes assessed (patient reported outcomes and experiences; care processes; costs); and the outcome data reported. Main findings of each study were summarized and presented in tables. Mean differences with 95% CI were calculated and presented in a summary of findings Table. A synthesis of the findings is presented in narrative format. Because of heterogeneity between studies regarding study designs and outcome measures used, meta-analysis was not conducted.

### Assessment of methodological quality

All authors were involved in assessing methodological quality of included studies working in randomly-assigned pairs to assess 3–4 studies each. Authors rotated through pairs to ensure consistency of decision-making. Differences in quality assessment scores were discussed by all authors and resolved using consensus. Quality assessment of the trial by Samsson et al. [24, 57, 58] was done by KG and JM, who had not been involved in that study. Assessment was made using a slightly modified version of the Downs and Black checklist (see Additional file 1) [59]. This checklist is appropriate for assessing study quality for RCTs and studies of other designs, and has better reliability and validity than other tools for studies of varied design [60, 61]. As previously reported [62] item 27 (study power) was modified for our review. The modified Downs and Black score ranges were assigned corresponding quality levels according to previously reported cut-offs [63]: excellent (26–28), good (20–25) fair (14–19) and poor (</ = 13). To reduce the risk of bias due to poor methodological quality, only studies with fair to excellent quality (i.e. score above 13) were included in the synthesis [64].

### Assessment of certainty of evidence

To assess confidence in the combined estimates of effect, the GRADE (Grading of Recommendations Assessment, Development and Evaluation) approach was applied for each outcome, using the following criteria: risk of bias, consistency, directness, precision, and reporting bias [65]. Two reviewers (KS and SB) performed assessments of certainty of evidence. A third reviewer (ML) was consulted if there was disagreement.

## Results

### Search results

**Fig. 1:**
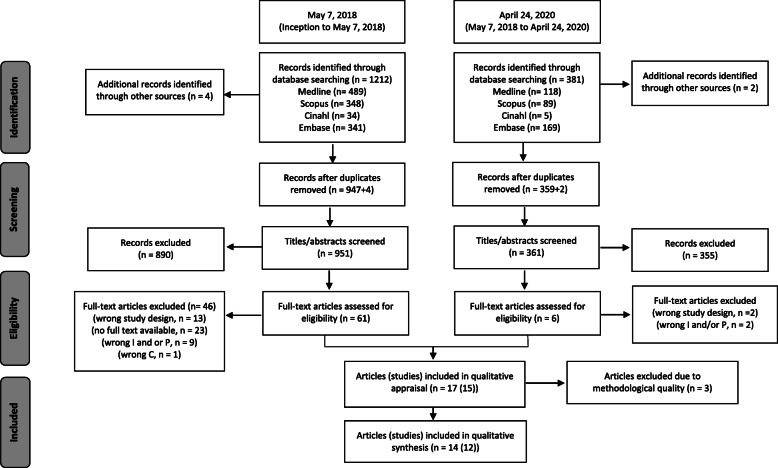
Flow diagram of selection process and search results. Adapted from Liberati et al. [51]

The search process and results are reported in a PRISMA flowchart [51], modified to account for the two literature searches (Fig. 1). The database searches yielded a total of 1593 citations, with six additional papers identified through reference list screening. After removing duplicates, 1312 papers remained, and after screening titles and abstracts as well as full text articles when necessary, 15 relevant studies were included, reported in 17 papers. After methodological quality assessment, three poor quality papers were excluded (see Additional file 2), leaving 12 relevant studies reported in 14 papers. Of the 12 included studies, five studies (reported in seven papers) [24, 57, 58, 66–69] had not been included in previous systematic reviews.

### Characteristics of included studies

Table 3 reports on the included studies; two RCTs (395 participants) and ten cohort studies (783 participants). These studies were published between 1999 and 2020, and were conducted in the United Kingdom [23, 70], Canada [20, 66, 69, 71, 72], Ireland [73] Australia [19, 67, 68], and Sweden [24, 57, 58]. Nine studies were conducted in orthopaedic hospital/outpatient departments/clinics, one in a tertiary care center, one in a knee screening clinic, and one in primary care.

**Table 3:** Characteristics of included studies

Authors, year	Country	Study design	Clinical setting	Aim/objective	Follow-up	Population, n	Intervention	Comparison	Outcomes
Ashmore et al., 2014	Ireland	Prospective audit	Knee screening clinic	To examine the proportion of patients managed independently by the ESP; analyse the accuracy of an ESP’s clinical diagnosis; calculate conversion rate to surgery of patients referred for orthopaedic consultation.		Patients with knee problems referred from GP or ED, *n* = 140 (subgroup analysis of IRR *n* = 57). 46% women, mean age 46.2 (SD 15.9, range 15–85) years.	PT triage (clinical assessment, diagnosis, appropriate management)	Medical imaging and/or surgery	Proportion of patients independently managed by the ESP; clinical diagnostic accuracy; surgical conversion rate
Daker-White et al., 1999 [23]	United Kingdom	RCT	Orthopaedic outpatient departments at two hospitals	To evaluate the effectiveness and cost effectiveness of specially trained PTs in assessment and management of defined referrals to hospital orthopaedic departments.	Mean (range) 5.5 (3–11) months	Patients with musculoskeletal problems referred from GP for orthopaedic consultation, *n* = 481 (complete data 191 + 192). 48% women in the PT group, 56% women in the OS group. Mean age (range) PT vs OS group 48.4 (17–87) years vs 48.6 (19–89) years.	Initial assessment + management by PT in extended role	Initial assessment + management by post-fellowship junior OS	Primary: Patient-centered measures of pain, functional disability, perceived handicap, self-efficacy, general health status, psychological status, health-related quality of life Secondary: Patient and GP satisfaction
Desmeules et al., 2013 [20]	Canada	Prospective inter-rater agreement study	Orthopaedic outpatient hospital clinic	To assess the diagnostic agreement of an APP compared to OS as well as to assess treatment concordance, healthcare resource use and patient satisfaction with this model.		Patients with hip and knee complaints referred from GP for initial consultation *n* = 120. 54% women, mean age 54.1 years.	PT assessment, diagnosis, triage recommendations (conservative or surgical management)	OS assessment, diagnosis, triage recommendations (conservative or surgical management)	Primary: inter-rater agreement for diagnosis, triage, treatment recommendations and imaging tests ordered. Secondary: Patient satisfaction
Dickens et al., 2003 [70]	United Kingdom	Prospective inter-rater agreement study	Outpatient knee clinic	To examine the ability of experienced PTs to make a correct diagnosis of patients presenting with acute knee injuries and to manage the diagnosis safely and effectively.		Patients with acute knee injuries, *n* = 50. 28% women, age 16–34 years.	PT assessment, diagnosis and management decisions (conservative/ surgical)	OS assessment, diagnosis and management decisions (conservative/ surgical)	Primary: Level of agreement between PT and OS. Secondary: Sensitivity, specificity and accuracy of clinicians’ diagnosis and management.
Jovic et al., 2019 [68]	Australia	Prospective inter-rater agreement study	Orthopaedic department, Hospital	To correlate the clinical skills of an ASP with the clinical standard of an OS across several domains, including diagnostic accuracy and treatment plan concordance.		Patients referred for hip or knee pain, *n* = 87. 51% women, mean age 67 (range 50–84).	ASP clinical examination including history and clinical assessment, diagnosis and treatment decision (surgical/ conservative/ further review needed).	OS assessment, diagnosis and treatment decision (surgical/conservative or further review needed).	Primary: diagnostic and treatment ability of the ASP Secondary: benefits on clinic efficiency and patient satisfaction
Lowry et al., 2020 [69]	Canada	Prospective cross-sectional concordance study	Orthopaedic outpatient clinic	To evaluate the diagnostic, surgical triage, and medical imaging agreement between APPs and OSs for the management of patients with shoulder pain; to compare patient satisfaction toward services provided by APPs and OSs.		Patients referred for shoulder pain, n = 50. 40% women, mean age 51.2 (yr +/−15.3).	APP clinical evaluation, diagnosis, further tests, treatment approach (conservative/ surgical/ referral to another medical specialist)	OS clinical evaluation, diagnosis, further tests, treatment approach (conservative/ surgical/ referral to another medical specialist)	Primary: diagnostic and treatment approach agreement. Secondary: imaging request agreement, patient satisfaction
MacKay et al., 2009 [71]	Canada	Prospective inter-rater agreement study	Hospital	To compare the clinical recommendations of specially trained PTs with those of an OS on appropriateness to be seen by an OS; and candidacy and willingness to undergo TJR; to examine their recommendations for non-surgical management and agreement on clinical diagnosis.		Patients referred for hip and knee problems with a diagnosis of arthritis, *n* = 62. 59% women, mean age 59.7, (range 33–85) years.	PT clinical assessment, diagnostics, treatment recommendations.	OS clinical assessment, diagnostics, treatment recommendations.	Primary: recommendations for OS consultation, recommendations for undergoing TJR. Secondary: recommendations for non-surgical management and agreement on clinical diagnosis
Marks et al., 2016 [67]	Australia	Blinded inter-rater agreement study	Orthopaedic outpatient setting	To establish the level of agreement between a PT and an OS regarding diagnosis, management and corticosteroid injection, in a representative sample of orthopaedic shoulder referrals		Patients with shoulder problems referred from GP to orthopaedic hospital, *n* = 274. 49% were women, mean age 57.9 (SD 13.0) years.	Clinical assessment by the PT, diagnosis, management plan	Clinical assessment by the OS, diagnosis, management plan	Primary: management and subacromial corticosteroid injection decisions. Secondary: level of diagnostic and investigation agreement.
Napier et al., 2013 [66]	Canada	Prospective observational trial	Orthopaedic clinic	To investigate the effectiveness of a PT triage service for orthopaedic surgery referrals from primary care physicians.		Patients with shoulder or knee problems referred from GP or ED with shoulder or knee (*n* = 45). 58% women, median age 47 (range 21–75) years.	PT assessment and categorisation as surgical or non-surgical (could be managed conservatively), needing further investigation or tests.	OS assessment and categorisation as surgical, non-surgical (could be managed conservatively), needing further investigation or tests.	Level of agreement, surgical conversion rate (SCR). Patient satisfaction
Oldmeadow et al., 2007 [19]	Australia	Prospective observational trial	Orthopaedic outpatient hospital department	To investigate the impact, quality and acceptability of a MSK screening clinic, provided by PT for patients referred to the outpatient orthopaedic department at a major metropolitan hospital		Patients with MSK related knee, shoulder or back pain (with or without leg-pain) referred from GPs, n = 45 (subgroup analysis *n* = 38). 60% women, mean age 58.6 (range 29–83) years.	PT screenings; comprehensive assessment, provisional diagnosis, management plan.	OS consultation; diagnoses and management decision	Level of agreement between the PT and OS on diagnoses and management decision. Levels of satisfaction (pt, GP, OS)
Razmjou, 2013 [72]	Canada	Prospective observational trial	Tertiary care centre	To examine the role of an APP with respect to agreement with OS on diagnosis and management of patients with shoulder problems; wait times; and satisfaction with care		Patients with shoulder complaints referred to a shoulder specialist, *n* = 100. 37% women, mean age 57 (SD 14; range 19–92) years. For satisfaction with care, *n* = 247 (similar age distribution).	PT patient history, assessment, diagnosis, management plan	OS patient history from PT, assessment, diagnosis, management plan	Agreement on clinical diagnosis, management (investigations, indications for surgery). Wait time Satisfaction.
Samsson et al., 2014, 2015, 2016 [24, 58, 57]	Sweden	RCT	Primary care	To evaluate PT-led orthopaedic triage of patients referred for orthopaedic consultation compared with standard practice in primary care; to report a long-term evaluation of patient-reported health-related quality of life, pain-related disability, and sick leave; to evaluate patients’ perceived quality of care.	3,6,12 months	Patients referred from GP with subacute or persistent MSK pain, *n* = 203. PT vs standard practice group 56% women, mean age 51 (range 18–67) years vs 55% women, mean age 53 (range 21–57) years.	PT-led orthopaedic triage; assessment, diagnosis, management pathway. Brief treatment.	OS assessment diagnosis, management pathway. Advice, prescriptions or injections, when appropriate.	Selection accuracy for orthopaedic intervention (i.e. surgical conversion rate) and other referrals, waiting time. PROMS; Self-reported health state (EuroQol VAS), health related quality of life (EQ-5D-3L) pain related disability (Pain Disability Index), sick leave. PREMS; perceived quality of care focusing on the caregivers’ medical-technical competence and identity-oriented approach; the extent to which patients’ expectations were met, patients’ intention to follow advice

*OS* orthopedic surgeon; *PT* physiotherapist; *APP* advanced practice physiotherapist; *CSP* clinical specialist physiotherapist; *ESP* extended scope physiotherapist; *pt.* patient; *GP* general practitioner; *IRR* interrater reliability; *MSK* musculoskeletal; *RCT* randomised controlled trial; *ED* emergency department; *QoL* quality of life; *TJR* Total joint replacement; *PROM* Patient reported outcome measure; *PREM* Patient reported experience measure; *SD* standard deviation; *SCR* surgical conversion rate

Participant numbers varied from 30 to 383 (age range 15–85 years, mean age (where available) ranging from 46.2 to 67 years). The majority of studies included patients with one or two problem areas; knee [70, 73], hip and knee [20, 68, 71], shoulder [67, 69, 72], shoulder or knee [66], shoulder, knee or spinal [19], with two studies having a broader inclusion of musculoskeletal or orthopaedic conditions [24, 57, 58, 67].

### Quality appraisal

Methodological quality was fair to good (see Table 4), with the two included RCTs (reported in four papers) assessed as having good methodological quality (range 19–22), and the ten cohort studies assessed as having fair to good methodological quality (range 14–23). Certainty of evidence was largely constrained. Two RCTs and three cohort studies had low to unclear risk of bias [23, 24, 57, 58, 67–69], and seven cohort studies had unclear risk of bias [19, 20, 66, 70–73]. One study [19] provided inadequate information and reported incomplete data. None of the included studies blinded healthcare professionals nor outcome assessors. Only one study [67] blinded patients to the profession of their assessors i.e., the patients did not know which clinician was the PT and which was the OS, and were unaware of their clinical decisions until all assessments had been completed. Almost all studies explicitly stated that they blinded assessors to each other’s findings [20, 66–73].

**Table 4:** Modified Downs and Blacks score

Paper	1	2	3	4	5	6	7	8	9	10	11	12	13	14	15	16	17	18	19	20	21	22	23	24	25	26	27	D&B score
Ashmore et al., 2014	y	y	y	y	y	y	y	n	y	n	y	y	n	n	n	y	y	y	y	y	y	y	n	n	n	y	n	18
Daker-White et al., 1999 [23]	y	y	y	y	p	y	y	n	n	y	n	n	y	n	n	y	y	y	y	y	y	y	y	y	n	n	y	19
Desmeules et al., 2013 [20]	y	y	y	y	y	y	y	n	n	y	n	n	n	n	n	y	y	y	y	y	y	y	n	n	n	y	y	18
Dickens et al., 2003 [70]	y	y	y	y	n	y	n	n	y	n	n	n	n	n	n	y	y	y	y	y	y	y	n	n	n	y	n	14
Jovic et al., 2019 [68]	y	y	y	y	y	y	y	n	y	n	n	n	n	n	n	y	y	y	y	y	y	y	n	n	n	y	y	18
Lowry et al., 2020 [69]	y	y	y	y	y	y	y	n	y	y	y	y	n	n	n	y	y	y	y	y	y	y	n	n	n	y	n	19
MacKay et al., 2009 [71]	y	y	y	y	y	y	y	n	y	n	n	n	y	n	n	y	y	y	y	y	y	y	n	n	n	y	y	19
Marks et al., 2016 [67]	y	y	y	y	p	y	y	n	y	y	y	y	y	y	y	y	n	y	y	y	y	y	n	y	n	y	y	23
Napier et al., 2013 [66]	y	y	y	n	n	y	y	n	y	n	n	n	n	n	n	y	y	y	y	y	y	y	n	n	n	y	y	15
Oldmeadow et al., 2007 [19]	y	y	y	y	n	y	y	n	y	n	n	n	y	n	n	y	n	y	y	y	y	y	n	n	n	n	n	15
Razmjou et al., 2013 [72]	y	y	y	y	p	y	y	n	y	y	n	n	y	n	n	y	n	y	y	y	y	y	n	n	n	y	n	16
Samsson et al., 2014 [24]	y	y	y	y	p	y	y	y	y	y	y	n	y	n	n	n	n	y	y	y	y	y	y	y	n	y	y	21
Samsson et al., 2015 [58]	y	y	y	y	p	y	y	y	n	y	y	y	y	n	n	n	y	y	y	y	y	y	y	y	n	y	y	22
Samsson et al., 2016 [57]	y	y	y	y	y	y	y	n	y	y	y	n	y	n	n	n	n	y	y	y	y	y	y	y	n	n	y	20

Criteria are based on the Downs and Black checklist (Additional file 1); y (yes) = criterion met, n (no) = criterion not met, p = criterion partially met. Item 5 has a maximum of 2 point, and all other items a maximum of 1 point. Maximum score for RCTs were 28 points, for non-randomised studies 25 points

A summary of findings of the included studies is presented in Table 5, and outcomes are discussed individually below.

**Table 5:** Summary of findings

Outcome	Absolute effect estimates (95% CI), PT-led triage vs standard care	No. of participants (studies)	Certainty in effect estimates (GRADE)	Conclusion
*Patient outcomes*				
Pain	MD in change −3.3 (−8.9 to 2.5); OR (6 mths) 0.9 (0.0 to 2.1)	519 (2 RCTs)	Low^b.c^	PT triage may result in little or no difference in pain compared with standard care.
Functional disability	MD in change 2.7 (−1.7 to 7.2); OR (6 mths) ranged from 1.4 to 2.0	318 (2 RCTs)	Low^b,c^	PT triage may result in little or no difference in functional disability compared with standard care.
Health state	MD in change ranged from −5.7 (−11.1 to −0.2) to 2.3 (−2.2 to 6.7)	524 (2 RCTs)	Very low^b,c.d^	It is uncertain whether PT triage results in any difference in health state compared with standard care.
Psychological status	MD in change −0.4 (−1.0 to 0.4); OR (6 mths) 1.9 (0.5 to 8.1)	512 (2 RCTs)	Low^b,c^	PT triage may result in little or no difference in psychological status compared with standard care.
Health-related quality of life	MD 0.0 (−0.1 to 1.1); OR (6 mths) ranged from 0.9 to 4.6.	537 (2 RCTs)	Low^b,c^	PT triage may result in little or no difference in psychological status compared with standard care.
Quality of care	Patient satisfaction: MD 3.0 (1.3 to 4.9) Quality from the Patient’s Perspective: MD 0.7^a^	549 (2 RCTs) 5 cohort studies also indicate high patient satisfaction	Moderate^,b^	PT triage probably slightly improves quality of care compared with standard care.
Sick leave	8 patients fewer; MD 74 days	203 (1 RCT)	Low^b,c^	PT triage may result in little or no difference in sick days compared with standard care.
*Process outcomes*				
Surgery conversion rate	Mean percentage difference 30% (11 to 49%).	203 (1 RCT) 3 cohort studies also present higher conversion rates	Moderate^,b^	PT triage probably results in higher surgery conversion rate than standard care.
Agreement on treatment approach (conservative or surgical)	Percentage agreement on treatment approach ranged from 70 to 93%.	910 (8 cohort studies)	Low	PT triage may have moderate to high agreement with standard care regarding treatment approach.
Investigation referrals	Mean percentage difference ranged from −27.6 to 32.8%	643 (2 RCTs) 2 cohort studies present an equivalent number of investigation referrals	Moderate^,b^	PT triage probably results in a reduction in investigation referrals compared with standard care.
Agreement on investigation referrals	Percentage agreement on investigation referrals ranged from 70 to 98%.	631 (5 cohort studies)	Low	PT triage may have varied agreement with standard care regarding investigation referrals.
Agreement on diagnosis	Percentage agreement on diagnosis ranged from 42 to 98%.	1062 (9 cohort studies)	Low	PT triage may have moderate to high agreement with standard care regarding diagnosis.
Waiting time	MD −9 days	203 (1 RCT) (1 cohort study also shows significantly shorter waiting time in PT group)	Moderate^,b^	PT triage probably reduces waiting time compared with standard care.
Cost effectiveness	MD in direct hospital costs per patient^a^ -£242	470 (1 RCT)	Moderate^,b^	PT triage is probably more cost effective than standard care.

*GRADE* Grading of Recommendations Assessment, Development and Evaluation; *CI* confidence interval; *MD* mean difference; *SD* standard deviation

^a^ 95% CI or dispersion measure not reported

^b^Downgraded one level due to serious risk of bias (mainly due to lack of blinding)

^c^Downgraded one level due to serious imprecision (large 95% CIs that include possible unfavourable effects)

^d^Downgraded one level due to serious inconsistency (effects in opposite directions)

GRADE Working Group grades of evidence

High certainty: We are very confident that the true effect lies close to that of the estimate of the effect

Moderate certainty: We are moderately confident in the effect estimate: The true effect is likely to be close to the estimate of the effect, but there is a possibility that it is substantially different

Low certainty: Our confidence in the effect estimate is limited: The true effect may be substantially different from the estimate of the effect

Very low certainty: We have very little confidence in the effect estimate: The true effect is likely to be substantially different from the estimate of effect

### Patient-reported outcomes

*Pain, disability, health state, psychological status*, and *health-related quality of life* were investigated in both RCTs [23, 58]. There were no significant differences in any outcome, between PT-led orthopaedic triage and standard care at three, six or 12 months’ follow-up (Table 6). For all outcomes, certainty was downgraded one level due to study limitations (mainly lack of blinding), and an additional level for serious imprecision (large 95% CIs that included possible unfavourable effects). For health state, certainty was further downgraded one level due to serious inconsistency (effects in opposite directions). Certainty of evidence was thus very low for health state, and low for all other patient-reported outcomes.

### Patient-reported experiences

These were reported in both RCTs [23, 57], as *patients’ perceptions of the quality of care received*, and their *satisfaction* with it (Table 6). Samsson et al. [57] reported significantly higher perceived quality of care for PT-led orthopaedic triage than for standard care, for all reported elements (e.g. caregivers’ medical technical competence, identity-oriented approach). Daker-White et al. [23] reported that patients were more satisfied after PT-led orthopaedic triage compared with standard care. Certainty of evidence for this outcome was moderate. The RCTs were downgraded one level due to study limitations, mainly lack of blinding. Four of the cohort studies also reported significant differences regarding patient satisfaction, favouring PT-led orthopaedic triage [19, 20, 66, 72] and one reporting no difference between groups [69].

**Table 6:** Patient-reported outcomes and experiences

Author, year	Patient-reported outcomes						Patient-reported experiences
	Pain	Functional disability	Health state	Psychological status	Health-related quality of life	Sick leave	Quality of care
Desmeules et al., 2013 [20]							Patient satisfaction significantly higher for the APP (93.2%, SD 13.5) than for the OS (86.1%, SD 23.3). MD 7.1 (95% CI 3.5 to 10.7; *p* < 0.001).
Daker-White et al., 1999 [23]	No significant differences between the groups. MD −3.3 (95% CI −8.9 to 2.5)	No significant differences between the groups. MD 2.7 (95% CI −1.7 to 7.2)	No significant differences between the groups. Thermometer score EQ. 5D 2.3 (95% CI − 2.2 to 6.7).	No significant differences between the groups. Anxiety: MD − 0.4 (95% CI − 1.0 to 0.4).	No significant differences between the groups. Health state score EQ. 5D 0.0 (95% CI − 0.1 to 0.1)		Patient dissatisfaction significantly lower in PT group, mean 28.0 (SEM 0.6), vs OS mean 31.0 (SEM 0.7) MD 3.0 (95% CI 1.3 to 4.9). Scale ranging 13–65, with 12 indicating greatest satisfaction.
Lowry et al., 2020 [69]							Patient satisfaction measured with the 9-item Visit-Specific Satisfaction Questionnaire was high, with no significant differences found between providers; 87.8 (SD 16.6) for the APP vs 86.9 (SD 19.1) for the OS (*p* = 0.697).
Napier et al., 2013 [66]							100% (45/45) of patients reported being satisfied or “very satisfied” (score 5) with overall care received from the PT (mean 4.87, range 4–5); 98% (44/45) reported being “satisfied” or “very satisfied” with advice/ education received from the PT (mean 4.67, range 3–5).
Oldmeadow et al., 2007 [19]							79% of patients reported being satisfied or very satisfied” with care they received from the PT. screening clinic (mean 1.4; range 1–4).
Razmjou et al., 2013 [72]							Measured with the Visit-Specific Satisfaction Instrument. Mean of total score was 649 (SD 71) for the APP and 606 (SD 103) for the OS (*p* = 0.004). For each question a significant difference in favour of the APP-led clinic.
Samsson et al., 2014 (I), 2015 (III), 2016 (II) [24, 58, 57]	No significant differences between the groups at 3, 6, 12 months. OR 3 m 0.8 (95% CI 0.0 to 21.1); 6 m 0.9 (95% CI 0.0 to 2.1); 12 m 0.7 (95% CI 0.0 to 16.5)	No significant differences between the groups at 3, 6, 12 months on PDI. Range OR 3 m 1.0 to 1.8, 6 m 1.4 to 2.0, 12 m 1.1 to 1.5	Significantly better health-state (EQ VAS) at 3 months after PT triage (mean difference − 5.7 (95% CI −11.1 to −0.2; *p* = 0.04)	No significant differences between the groups at 3,6, 12 months. Anxiety: OR 3 m 0.9 (95% CI 0.3 to 3.1); 6 m 1.9 (95% CI 0.5 to 8.1); 12 m 1.6 (95% CI 0.5 to 5.2)	No significant differences between the groups at 3, 6, 12 months on EQ. 5D. Range OR 3 m 0.8 to 1.8; 6 m 0.9 to 4.6; 12 m 0.7 to 1.8	No significant differences; 7 patients in the PT group, mean days 146 (SD 128), 15 patients in the OS group, mean days 72 (SD 81) (*p* = 0.113)	Measured with the Quality from the Patient’s Perspective; “do not agree at all” to “completely agree” (score 4). Significantly higher perceived quality of care after PT triage compared with OS with regard to receiving best possible examination and treatment, mean 3.5 (Q1 3; Q3 4) vs 2.9 (Q1 2; Q3 4) (*p* < 0.001), as well as information about examination and treatment, mean 3.6 (Q1 3; Q3 4) vs 3.0 (Q1 2; Q3 4) (*p* < 0.001), results, mean 3.4 (Q1 3; Q3 4) vs 2.7 (Q1 2; Q3 4) (*p* < 0.001), self-care, mean 3.5 (Q1 3; Q3 4) vs 2.9 (Q1 2; Q3 4) (*p* < 0.001), caregivers’ understanding, mean 3.8 (Q1 4; Q3 4) vs 3.1 (Q1 2.5; Q3 4) (*p* < 0.001), respect, mean 3.9 (Q1 4; Q3 4) vs 3.4 (Q1 3; Q3 4) (*p* < 0.001), commitment, mean 3.9 (Q1 4; Q3 4) vs 3.0 (Q1 2; Q3 4) (*p* < 0.001), and participation in decision-making, mean 3.6 (Q1 3; Q3 4) vs 3.2 (Q1 3; Q3 4) (*p* = 0.01). Expectations were met to a significantly higher extent after PT triage, mean 4.3 (Q1 4; Q3 5) vs 3.7 (Q1 3; Q3 4) for OS (*p* < 0.001), ranging from 1 (not at all) to 5 (to a very large extent). Intention to follow advice and instructions received was significantly greater after PT triage, mean 2.8 (Q1 3; Q3 3) vs 2.6 (Q1 2; Q3 3) for OS (*p* = 0.019), ranging from 1 (no) to 3 (yes completely).

*PT* physiotherapist; *APP* Advanced Practice Physiotherapist; *OS* orthopedic surgeon; *GP* general practitioner; *MD* mean difference; *SD* standard deviation; *CI* confidence interval; *OR* Odds ratio; *EQ. 5D* EuroQol 5D, *PDI* Pain disability index; *Q1; Q3* quartile range 1; 3

### Sick leave

(Days off work) was investigated in one RCT [58], which reported no differences between study arms (Table 6). Certainty of evidence was assessed as low; this having been downgraded one level due to study limitations (mainly due to lack of blinding) and an additional level due to serious imprecision (large 95% CIs that possibly included unfavorable effects).

### Process outcomes

Inter-rater reliability of decision-making, care processes and costs are reported in Table 7.

**Table 7:** Outcomes related to care processes and cost effectiveness

Author, year	Surgical conversion rate (SCR) /selection accuracy	Agreement for treatment approach (conservative or surgical)	Investigation referrals	Agreement on investigation referral	Agreement on diagnosis	Waiting time	Cost effectiveness
Ashmore et al., 2014	SCR of 84% (42/50)				Raw agreement, 88% (κ = 0.795;95% CI, 0.58–1.00) between ESP and medical imaging or surgery.		
Daker-White et al., 1999 [23]			A greater proportion of PTs ordered no investigations at all (47.5% vs 14.7%; *p* **<** 0.001). A smaller proportion of PTs ordered plain x-rays (13.9% vs 41.5%; *p* < 0.001). No significant differences in other diagnostic tests ordered.				No significant differences in direct costs to the patient or NHS primary care costs. Direct hospital costs were significantly lower in the PT arm (mean cost per patient £256 vs £498 *p* < 0.001), as PTs were less likely to order radiographs and to refer patients for orthopaedic surgery.
Desmeules et al., 2013 [20]		Raw agreement, 88%, between APP and OS (κ = 0.77; 95% CI:0.65–0.88)	No significant differences when ordering any type of imaging tests between APP and OS (*p* = 0.113).	General inter-rater agreement (κ = 0.65; 95% CI:0.52–0.79), for X-rays only (κ = 0.48; 95% CI:0.33–0.64)	Raw agreement, 88%, (κ = 0.86, 95% CI:0.80–0.93) between APP and OS		
Dickens et al., 2003 [70]					Raw agreement among all 3 clinicians: 76.5% (13/17). Correct diagnosis by the orthopaedic consultant 92%, PT 1 84%, PT 2 80%. Diagnostic accuracy for various types of injuries ranged from 96 to 100% for the OS, and from 94 to 98% for the PTs.		
Jovic et al., 2019 [68]	SCR 78% with ASP-led service; with prior orthopaedic-led model 38%.	Inter-rater agreement between ASP and OS on treatment (κ = 0.75; 95% CI 0.62–0.89).		Inter-rater agreement between ASP and OS (κ = 0.93; 95% CI 0.87–1.00).			
Lowry et al., 2020 [69]		Raw agreement, 70%, on surgery between PT and OS (κ **=** 0.46; 95% CI 0.21–0.71).	No significant differences in terms of frequency of medical imaging requests were found between Pt and OS; x-rays (*p* = 0.338), MRI (*p* = 0.799), other tests (*p* = 0.400).	Raw agreement, 70%, between PT and OS (κ **=** 0.42; 95% CI 0.19–0.66).	Raw agreement, 86%, between PT and OS (κ **=** 0.80; 95% CI 0.67–0.93).		
MacKay et al. 2009 [71]		Raw agreement, 85.5% (53/62) on surgery between PT and OS (κ = 0.70)			Raw agreement 69% between PT and OS.		
Marks et al., 2016 [67]		Raw agreement, 94%, between PT and OS (AC1 = 0.93; 95% CI 0.90–0.93)		Raw agreement, 88%, between PT and OS (AC1 = 0.87; 95% CI 0.83–0.91)	Raw agreement, 74%, between PT and OS (AC1 = 0.72; 95%CI 0.66–0.78)		
Napier et al., 2013 [66]	SCR referred by the APP 91%, vs 22% of patients referred by a GP or ED physician.	Raw agreement, 84.4% between APP and OS (κ **=** 0.77; 95% CI, 0.60–0.94).					
Oldmeadow et al., 2007 [19]		Raw agreement, 74%, between PT and OS on management decisions (κ = 0.38: 95% CI 0.13–0.63).					
Razmjou et al., 2013 [72]		Raw agreement 88% between APP and OS for surgery. The APP tended to recommend surgery more often than the OS, 65% vs 55%, (κ = 0.75; 95% CI 0.62 to 0.88).		Raw agreement 97% on x-rays between APP and OS (κ = 0.91; 95% CI 0.81 to 1.00).	Raw agreement on major diagnostic categories between APP and OS varied from 84 to 98% (κ = 0.68 to 0.94)	Significantly shorter waiting time for APP assessment than for OS assessment at all three time points (Wilcoxon 6.20, 5.92 and 5.41, *p* < 0.001)	
Samsson et al., 2014, 2015, 2016 [24, 58, 57]	Significantly higher SCR with PT triage, 55%, vs standard practice 25%; difference 30% (95% CI 11 to 49%), *p* = 0.002).		Significantly lower proportion of investigations ordered by the PT (17% vs 29%; difference − 12% (95% CI −23 to 0.6%), *p* = 0.039.			Significantly fewer days in PT group 19 (SD 12) vs 28 (SD 14) days in the standard practice group (*p* < 0.001)	

*SCR* surgery conversion rate; *PT* physiotherapist; *APP* advanced practice physiotherapist; *ESP* extended scope physiotherapist; *CSP* clinical specialist physiotherapist; *OS* orthopedic surgeon; *GP* general practitioner; *CI* confidence interval; *SD* standard deviation; *NHS* national healthcare services; *TJR* total joint replacement; *AC1* Gwets first order agreement coefficient

*Surgery conversion rate (SCR)* refers to the number of patients who proceeded to surgery following assessment, with the decision of the OS considered the gold standard. Surgery conversion rate was assessed in one RCT [24] and three observational studies [66, 68, 73]. Certainty of evidence was moderate, as the RCTs were downgraded one level due to study limitations (mainly lack of blinding). Samsson et al. [24] reported a higher SCR after PT-led orthopaedic triage (55%, compared with 25% in standard care) (*p* = 0.002). The observational studies reported higher SCR rates: Napier et al. [66] reported 91% after PT-led orthopaedic triage compared with 22% for standard care; Jovic et al. [68] 78% compared with 38%, and Ashmore et al. [73] reported a conversion to surgery rate of 84% with PT led orthopaedic triage.

Good *agreement between PTs and other medical professionals* *regarding treatment approach (conservative or surgical)* is relevant to the safety of PT-led orthopaedic triage, as it is important that PTs do not offer a lesser standard of assessment and decision-making than OS. Comparison between treatment approach made by PTs and OS was reported in seven cohort studies [19, 20, 67–69, 71, 72], indicating overall strong percentage agreement, ranging from 70 to 94%, and inter-rater agreement (κ-values ranging from 0.38 to 0.77 and AC1 = 0.93). However, the certainty of evidence was low due to the study design.

*Referral for investigations* was evaluated in the two RCTs [23, 24] and in two cohort studies [20, 69] with mean percentage difference in referral proportions between PTs and OS ranging from − 27.6 to 32.8%. Samsson and Larsson [24] reported a significantly lower proportion of patients referred for investigations for PT-led OT compared with standard care (17% vs 29%; difference − 12% [95% CI − 23 to 0.6%], *p* = 0.039). Daker-White et al. [23] reported that a higher proportion of PTs ordered no investigations at all (48% vs 15%; *p* **<** 0.001) and fewer plain X-rays (14% vs 41%; *p* < 0.001) compared with standard care. Certainty of evidence was moderate. The RCTs were downgraded one level due to study limitations, mainly lack of blinding. The cohort studies reported equal rate of investigations between PTs and OSs [20, 69].

*Agreement on referral for investigations* was reported in five cohort studies [20, 67–69, 72], overall strong percentage agreement (70 to 97%) and inter-rater agreement (κ-values ranging from 0.42 to 0.93 and AC1 = 0.87). However, certainty of evidence was low due to study designs.

*Agreement on diagnosis* between PTs and OS or imaging/surgery findings was investigated in seven cohort studies [20, 67, 69–73] with overall good percentage agreement (ranging from 72 to 98%) and inter-rater agreement (κ-values ranging from 0.68 to 0.94 and AC1 = 0.72. However, certainty of evidence was low due to the study designs.

*Waiting time* was investigated in one RCT [24], reporting significantly shorter waiting times to PT-led orthopaedic triage; 19 (SD 12) compared with 28 (SD 14) days for standard care (*p* < 0.001). One cohort study [72] also reported significantly shorter waiting times with PT-led OT (*p* < 0.001). Certainty of evidence was assessed as moderate. The RCT was downgraded one level due to study limitations, mainly lack of blinding.

*Cost effectiveness* of PT-led orthopaedic triage compared with standard care was investigated in one RCT [23] . No significant differences were found in terms of direct costs to the patient, or in organisational (NHS primary care) costs. Direct hospital costs were significantly lower in the PT-led orthopaedic triage arm (mean cost per patient £256 vs £498 in the standard care arm (*p* < 0.001)), as PTs were less likely to order radiographs or refer patients for orthopaedic surgery. Certainty of evidence was moderate (downgraded one level due to study limitations, mainly lack of blinding).

*Adverse events* were not reported as having occurred in any study.

## Discussion

This review updates the current body of secondary evidence on the impact of PT-led orthopaedic triage compared with standard OS care, for people with musculoskeletal disorders. We identified 12 studies, five of which had not been included in previous reviews [47–50]. Our review found evidence of moderate certainty that PT-led orthopaedic triage results in higher surgery conversion rates, reduces investigation referral rates and waiting times for orthopaedic consultation, and improves quality of care (patient satisfaction). We found low-certainty evidence for moderate to high agreement between PT-led orthopaedic triage and standard care in diagnosis, treatment and investigation approaches. For patient-reported outcomes, there is low-certainty evidence for no difference between PT-led orthopaedic triage and standard care. Taken together, our findings suggest that PTs are equally effective as OSs in assessing and managing patients referred for orthopaedic consultation, although the low number of studies and the heterogeneity in methodological quality limits our ability to draw firm conclusions.

The evidence base for the effectiveness of PT-led orthopaedic triage is growing, particularly for the assessed process outcomes. Our findings build on the findings of the earlier reviews [47–50], which concluded low certainty of evidence for the positive impact on both patient and process outcomes of PT-led orthopaedic triage. With the addition of the recent studies identified in our review, the certainty of evidence for the effect on process outcomes has increased, whilst the evidence for PT-led orthopaedic triage impact on patient health outcomes and patient satisfaction with quality of care remains low.

Considering that PTs cost the health system approximately half as much as an OS [27], and as they reduced rates of referrals for investigation [23, 24], PT-led triage could be a cost effective way to address growing waiting lists and ensure that patients needing an orthopaedic consultation receive it in a more timely manner. The significantly shortened waiting times using a PT-led triage model [24, 72] could also have a further impact on economic parameters. A recent scoping review [74] suggests that waiting has a negative effect on patient, institution and societal costs, although the body of evidence was scant. Furthermore, Morris et al. [75] suggest that prolonged waiting time for an orthopaedic consultation may impact quality of life.

A higher conversion to surgery rate suggests that the triage process undertaken by PTs provides patients in need of surgery with an expedited path to requisite treatment, not that an increased number of surgeries are performed. This review generally indicates that PTs can provide earlier assessment than an OS, and earlier identification of patients requiring orthopaedic interventions may facilitate these patients being fast-tracked to further investigations and an OS consultation. Furthermore, PT-triage can offer patients that may not require an OS assessment in the first instance alternative (conservative) care options. An additional benefit is that PTs working with triage assessment tend to give patients advice on conservative management and self-care, e.g. home exercise [20, 57], which may contribute to quicker recovery and further reduce the need for additional healthcare visits. Fennely et al. [76] explored patients’ experience after PT-led triage and found that that patients valued that the PT listened to them, and that they could provide more specific advice regarding self-management, a finding also identified by Samsson et al. [57]. Both studies also found that patients had confidence in the PTs’ professional ability.

However, there are limitations when evaluating process outcomes with this model of care. The lower rate of investigation after PT-led triage which is perceived as a good outcome, could in fact be a limitation, as some pathology might be overlooked. Furthermore, the PTs might be biased towards avoiding both investigation and surgery as an intervention, whereas the OS might be biased towards performing surgery. One of the reasons for implementing PT-led triage has been to reduce waiting times, and the findings in this review of reduced waiting times with PT-led triage is a factor that could change with time, as demand increases. Furthermore, it could be argued that costs might increase, due to many patients being referred for further physiotherapy after PT-led triage [24]. Another issue with this model of care is the PTs’ advanced role, allowing professional development but also entailing greater responsibility [77]. This increased responsibility should be reflected in salaries, and with that, costs might increase.

The varying titles and positions of PTs, such as advanced practice, extended scope or experienced PTs, and the varying level of training and tasks undertaken potentially constrain comparison between studies. In most countries no formal training exists and availability of experienced PTs might be scarce. Furthermore, PTs working in this role have expressed a lack of formal education [78]. In order to standardise this model of care internationally and improve reporting, a framework for competencies and education standards should be used, such as the one provided recently by Fennelly et al. [79].

A limitation when evaluating this model of care is the lack of consistency amongst studies in patient-reported outcomes, or ways in which patient satisfaction was measured. This limited comparison between studies and review conclusions. Fennely et al. [80] also identified the lack of measurement of global improvement, psychological well-being and/or work ability in their recent systematic review on the use of outcome measures in advanced PT practice, and that although patient satisfaction was frequently measured, non-standardised, locally-devised tools were used.

### Strengths and limitations of this review

We modified the PROSPERO-registered protocol once the review had commenced to include prospective comparative studies, because of the paucity of RCTs. Although studies published in any language were eligible for inclusion, only English-language articles were identified. Important limitations of the review are that we only identified two RCTs, and that risk of bias to varying extent were seen in all included studies. Lack of blinding was a problem in all studies.

Definition of terms was problematic. The term ‘triage’ may have been interpreted differently by different researchers. The distinction between triage, screening, and assessment is subtle. We included all these terms in our search strategy, but the development and adoption of consistent terminology and operational definitions for these forms of assessment could facilitate improved understanding among researchers, clinicians and policy makers. Whilst standard care appears to be a relatively vague term, in our review as in all studies included in the review, standard care was considered to be referral from a GP to, and assessment by, an OS.

The included studies involved patients with varied musculoskeletal conditions [24, 57, 58, 67] or patients with one or two problem areas; knee [70, 73], shoulder and knee [66], hip and knee [20, 68, 71] and shoulder [67, 69, 72], shoulder, knee or spinal [19] respectively. Thus, the conditions included in this review are heterogeneous, and the significance of differences between PT-led orthopaedic triage and standard care may have been confounded by different care needs of patients with different conditions.

The Downs and Black checklist used in this review is appropriate for assessing study quality for both RCTs and cohort studies, and has better reliability and validity than other tools for studies of varied design [60, 61]. However, we found it to be less applicable to the cohort studies than to the RCTs. In hindsight, the checklist could have been modified so that the items addressing for example follow-up and randomisation could be scored ‘not applicable’ in cohort studies (thus adjusting the denominator). Therefore, methodological quality of the assessed papers in this review might be lower than previously reported. In order to avoid assessor bias, the quality assessment of the trial by Samsson et al. [24, 57, 58] was done by the authors KG and JM, who had not been involved in that study. Lastly, there is a risk for publications bias; mean differences were higher in the smaller studies included, suggesting that our overall estimate is potentially overestimated.

### Future research

There is a need for more, and better quality, primary studies, particularly RCTs, so that the evidence base will have less bias and more certainty. Future studies should be innovative about how patients, clinicians and evaluators can be blinded to allocation to intervention arms. This would improve methodological quality scores and increase the believability of study findings.

## Conclusions

There is a growing body of evidence, of low to moderate certainty, that PT-led orthopaedic triage and OS make similar diagnosis, treatment and investigation decisions for patients with musculoskeletal disorders, and that patients are equally or more satisfied with quality or care. PT-led orthopaedic triage is safe, less expensive than OS, and effective in triaging patients on orthopaedic waiting lists to ensure that patients are directed to the most appropriate care as quickly as possible. Consistent with previous reviews, the current body of evidence is limited by study volume, design and quality, as well as heterogenous outcome measures. More research of higher quality is required to investigate the impact of PT-led orthopaedic triage on patient reported outcomes and experiences, work ability, sick leave, cost-effectiveness, and length of waiting time. Rigorous RCTs are needed to increase certainty of the evidence.

## Supplementary information

**Additional file 1.** Modified Downs and Black checklist.

**Additional file 2.** Excluded publications.

**Additional file 3.** PRISMA checklist.

## Data Availability

This systematic review is solely based on material published in the included original studies.

## Abbreviations

GP: General practitioner
OS: Orthopaedic surgeon
PT: Physiotherapist
RCT: Randomised controlled trial
SCR: Surgery conversion rate

## References

[1] Murray CJL, Vos T, Lozano R, Naghavi M, Flaxman AD, Michaud C, Ezzati M, Shibuya K, Salomon JA, Abdalla S (2012). Disability-adjusted life years (DALYs) for 291 diseases and injuries in 21 regions, 1990-2010: a systematic analysis for the global burden of disease study 2010. Lancet.

[2] Cross M, Smith E, Hoy D, Nolte S, Ackerman I, Fransen M, Bridgett L, Williams S, Guillemin F, Hill CL (2014). The global burden of hip and knee osteoarthritis: estimates from the global burden of disease 2010 study. Ann Rheum Dis.

[3] Hoy D, March L, Brooks P, Blyth F, Woolf A, Bain C, Williams G, Smith E, Vos T, Barendregt J (2014). The global burden of low back pain: estimates from the global burden of disease 2010 study. Ann Rheum Dis.

[4] Smith E, Hoy DG, Cross M, Vos T, Naghavi M, Buchbinder R, Woolf AD, March L (2014). The global burden of other musculoskeletal disorders: estimates from the global burden of disease 2010 study. Ann Rheum Dis.

[5] Vos T, Allen C, Arora M, Barber RM, Brown A, Carter A, Casey DC, Charlson FJ, Chen AZ, Coggeshall M (2016). Global, regional, and national incidence, prevalence, and years lived with disability for 310 diseases and injuries, 1990–2015: a systematic analysis for the global burden of disease study 2015. Lancet.

[6] March L, Smith EUR, Hoy DG, Cross MJ, Sanchez-Riera L, Blyth F, Buchbinder R, Vos T, Woolf AD (2014). Burden of disability due to musculoskeletal (MSK) disorders. Best Pract Res Cl Rh.

[7] Jordan KP, Kadam UT, Hayward R, Porcheret M, Young C, Croft P (2010). Annual consultation prevalence of regional musculoskeletal problems in primary care: An observational study. BMC Musculoskelet Disord.

[8] Månsson J, Nilsson G, Strender LE, Björkelund C (2011). Reasons for encounters, investigations, referrals, diagnoses and treatments in general practice in Sweden-a multicentre pilot study using electronic patient records. Eur J Gen Pract.

[9] Jordan KP, Jóud A, Bergknut C, Croft P, Edwards JJ, Peat G, Petersson IF, Turkiewicz A, Wilkie R, Englund M (2014). International comparisons of the consultation prevalence of musculoskeletal conditions using population-based healthcare data from England and Sweden. Ann Rheum Dis.

[10] The Muscoloskeletal Services Framework - A Joint Responsibility: Doing it differently [http://webarchive.nationalarchives.gov.uk/20130107105354/http://www.dh.gov.uk/prod_consum_dh/groups/dh_digitalassets/@dh/@en/documents/digitalasset/dh_4138412.pdf] Accessed 13 Nov 2019.

[11] MacKay C, Canizares M, Davis AM, Badley EM (2010). Health care utilization for musculoskeletal disorders. Arthritis Care Res.

[12] Canizares M, MacKay C, Davis AM, Mahomed N, Badley EM (2009). A population-based study of ambulatory and surgical services provided by orthopaedic surgeons for musculoskeletal conditions. BMC Health Serv Res.

[13] Curtis AJ, Russell COH, Stoelwinder JU, McNeil JJ (2010). Waiting lists and elective surgery: ordering the queue. Med J Aust.

[14] Hoogeboom TJ, van den Ende CHM, van der Sluis G, Elings J, Dronkers JJ, Aiken AB, van Meeteren NLU (2009). The impact of waiting for total joint replacement on pain and functional status: a systematic review. Osteoarth Cartilage.

[15] Brinker MR, O'Connor DP, Pierce P, Woods GW, Elliott MN (2002). Utilization of orthopaedic services in a capitated population. J Bone Joint Surg - Series A.

[16] Menzies RD, Young RA (2012). Referrals from a primary care-based sports medicine department to an orthopaedic department: a retrospective cohort study. Br J Sports Med.

[17] McHugh GA, Campbell M, Luker KA (2011). GP referral of patients with osteoarthritis for consideration of total joint replacement: a longitudinal study. Br J Gen Pract.

[18] Saleh KJ, Bozic KJ, Graham DB, Shaha SH, Swiontkowski MF, Wright JG, Robinson BS, Novicoff WM (2013). Quality in Orthopaedic surgery—an international perspective: AOA critical issues. J Bone Joint Surg.

[19] Oldmeadow LB, Bedi HS, Burch HT, Smith JS, Leahy ES, Goldwasser M (2007). Experienced physiotherapists as gatekeepers to hospital orthopaedic outpatient care. Med J Aust.

[20] Desmeules F, Toliopoulos P, Roy JS, Woodhouse LJ, Lacelle M, Leroux M, Girard S, Feldman DE, Fernandes JC (2013). Validation of an advanced practice physiotherapy model of care in an orthopaedic outpatient clinic. BMC Musculoskelet Disord.

[21] Robarts S, Kennedy D, MacLeod AM, Findlay H, Gollish J (2008). A framework for the development and implementation of an advanced practice role for physiotherapists that improves access and quality of care for patients. Healthc Q.

[22] Fennelly O, Blake C, Fitzgerald O, Breen R, Ashton J, Brennan A, Caffrey A, Desmeules F, Cunningham C (2018). Advanced practice physiotherapy-led triage in Irish orthopaedic and rheumatology services: National data audit. BMC Musculoskelet Disord.

[23] Daker-White G, Carr AJ, Harvey I, Woolhead G, Bannister G, Nelson I, Kammerling M (1999). A randomised controlled trial. Shifting boundaries of doctors and physiotherapists in orthopaedic outpatient departments. J Epidemiol Community Health.

[24] Samsson K, Larsson MEH (2014). Physiotherapy screening of patients referred for orthopaedic consultation in primary healthcare - a randomised controlled trial. Man Ther.

[25] Desmeules F, Roy JS, Macdermid JC, Champagne F, Hinse O, Woodhouse LJ (2012). Advanced practice physiotherapy in patients with musculoskeletal disorders: a systematic review. BMC Musculoskelet Disord.

[26] Morris JH, James RE, Davey R, Waddington G (2014). What is orthopaedic triage? A systematic review. J Eval Clin Pract.

[27] Morris J, Grimmer-Somers K, Kumar S, Murphy K, Gilmore L, Ashman B, Perera C, Vine K, Coulter C (2011). Effectiveness of a physiotherapy-initiated telephone triage of orthopedic waitlist patients. Patient Relat Outcome Meas.

[28] Guidance to using the pay tool [https://www.nhsemployers.org/-/media/Employers/Documents/Pay-and-reward/2018-contract-refresh/Spine-points-at-31-March-2018.pdf] Accessed 14 of Jan 2020.

[29] NHS Doctor’s Pay Scales in the UK - Explained [https://www.imgconnect.co.uk/news/2019/06/nhs-doctors-pay-scales-in-the-uk-explained/59#Consultants] Accessed 15 Apr 2020.

[30] Pay Scale Orthopaedic Surgeon Salary Australia [https://www.payscale.com/research/AU/Job=Orthopedic_Surgeon/Salary] Accessed 15 Apr 2020.

[31] Salary Expert Orthopedic Surgeon Salary Australia [https://salaryexpert.com/salary/job/orthopedic-surgeon/australia] Accessed 15 April 2020.

[32] ACT Public Sector Health Professional Enterprise Agreement 2018–2021 [https://www.health.act.gov.au/sites/default/files/2019-08/Health-Professional-Enterprise-Agreement-2018-2021.pdf] Accessed 15 Apr 2020.

[33] Nuevoo Physiotherapist Salary in Canada [https://neuvoo.ca/salary/?job=Physiotherapist] Accessed 15 Apr 2020.

[34] PayScale Orthopedic Surgeon Salary in Canada [https://www.payscale.com/research/CA/Job=Orthopedic_Surgeon/Salary] Accessed 15 Apr 2020.

[35] Average monthly salary by occupation, 2019 [https://www.scb.se/en/finding-statistics/statistics-by-subject-area/labour-market/wages-salaries-and-labour-costs/wage-and-salary-structures-and-employment-in-the-primary-municipalities/pong/tables-and-graphs/average-monthly-salary-by-occupation/] Accessed 15 Apr 2020.

[36] Lönestatistik ortopedläkare [https://yrkeskollen.se/lonestatistik/ortoped/] Accessed 15 Apr 2020.

[37] Björnsdóttir SV, Jónsson SH, Valdimarsdóttir UA (2013). Functional limitations and physical symptoms of individuals with chronic pain. Scand J Rheumatol.

[38] Bingefors K, Isacson D (2004). Epidemiology, co-morbidity, and impact on health-related quality of life of self-reported headache and musculoskeletal pain - a gender perspective. Eur J Pain.

[39] Picavet HSJ, Schouten JSAG (2003). Musculoskeletal pain in the Netherlands: prevalences, consequences and risk groups, the DMC (3)-study. Pain.

[40] Landmark T, Romundstad P, Dale O, Borchgrevink PC, Vatten L, Kaasa S (2013). Chronic pain: one year prevalence and associated characteristics (the HUNT pain study). Scand J Pain.

[41] Kroenke K, Outcalt S, Krebs E, Bair MJ, Wu J, Chumbler N, Yu Z (2013). Association between anxiety, health-related quality of life and functional impairment in primary care patients with chronic pain. Gen Hosp Psychiatry.

[42] Ackerman IN, Ademi Z, Osborne RH, Liew D (2013). Comparison of health-related quality of life, work status, and health care utilization and costs according to hip and knee joint disease severity: a national Australian study. Phys Ther.

[43] Kennedy DM, Robarts S, Woodhouse LJ (2010). Patients are satisfied with advanced practice physiotherapists in a role traditionally performed by orthopaedic surgeons. Physiother Can.

[44] Leviton LC, Melichar L (2016). Balancing stakeholder needs in the evaluation of healthcare quality improvement. BMJ Quality &amp;amp; Safety.

[45] Crossing the quality chasm: a new health system for the 21st century [http://www.nationalacademies.org/hmd/Reports/2001/Crossing-the-Quality-Chasm-A-New-Health-System-for-the-21st-Century.aspx] Accessed 20 Aug 2016. 25057539

[46] Quality assurance standards of physiotherapy service delivery [http://www.csp.org.uk/publications/quality-assurance-standards] Accessed 22 Aug 2013.

[47] Joseph C, Morrissey D, Abdur-Rahman M, Hussenbux A, Barton C (2014). Musculoskeletal triage: a mixed methods study, integrating systematic review with expert and patient perspectives. Physiotherapy (United Kingdom).

[48] Hussenbux A, Morrissey D, Joseph C, McClellan CM (2015). Intermediate care pathways for musculoskeletal conditions - are they working? A systematic review. Physiotherapy (United Kingdom).

[49] Oakley C, Shacklady C (2015). The clinical effectiveness of the extended-scope physiotherapist role in musculoskeletal triage: a systematic review. Musculoskeletal Care.

[50] McEvoy C, Wiles L, Bernhardsson S, Grimmer K (2017). Triage for Patients with Spinal Complaints: A Systematic Review of the Literature. Physiother Res Int.

[51] Liberati A, Altman DG, Tetzlaff J, Mulrow C, Gøtzsche PC, Ioannidis JPA, Clarke M, Devereaux PJ, Kleijnen J, Moher D (2009). The PRISMA statement for reporting systematic reviews and meta-analyses of studies that evaluate health care interventions: Explanation and elaboration. PLoS Med.

[52] Cochrane handbook for systematic reviews of interventions, version 6.0 [https://training.cochrane.org/handbook] Accessed 7 Nov 2019.

[53] Akobeng AK (2005). Principles of evidence based medicine. Arch Dis Child.

[54] Schardt C, Adams MB, Owens T, Keitz S, Fontelo P: Utilization of the PICO framework to improve searching PubMed for clinical questions. BMC Med Inform Decis Mak 2007, 7, 16(. 10.1186/1472-6947-7-16 PMC1904193 17573961

[55] Systematic Reviews: CRD’s Guidance for Undertaking Reviews in Health Care [https://www.york.ac.uk/media/crd/Systematic_Reviews.pdf] Accessed Nov 9 2019.

[56] Ouzzani M, Hammady H, Fedorowicz Z, Elmagarmid A (2016). Rayyan-a web and mobile app for systematic reviews. Syst Rev.

[57] Samsson KS, Bernhardsson S, Larsson ME (2016). Perceived quality of physiotherapist-led orthopaedic triage compared with standard practice in primary care: a randomised controlled trial. BMC Musculoskelet Disord.

[58] Samsson KS, Larsson MEH (2015). Physiotherapy triage assessment of patients referred for orthopaedic consultation - long-term follow-up of health-related quality of life, pain-related disability and sick leave. Man Ther.

[59] Downs SH, Black N (1998). The feasibility of creating a checklist for the assessment of the methodological quality both of randomised and non-randomised studies of health care interventions. J Epidemiol Community Health.

[60] Deeks JJ, Dinnes J, D'Amico R, Sowden AJ, Sakarovitch C, Song F, Petticrew M, Altman DG (2003). Evaluating non-randomised intervention studies. Health Technol Assess.

[61] Hootman JM, Driban JB, Sitler MR, Harris KP, Cattano NM (2011). Reliability and validity of three quality rating instruments for systematic reviews of observational studies. Res Synth Methods.

[62] Ojha HA, Snyder RS, Davenport TE (2014). Direct access compared with referred physical therapy episodes of care: a systematic review. Phys Ther.

[63] Hooper P, Jutai JW, Strong G, Russell-Minda E (2008). Age-related macular degeneration and low-vision rehabilitation: a systematic review. Can J Ophthalmol.

[64] Navarro CM, Brolund A, Ekholm C, Heintz E, Ekström EH, Josefsson PO, Leander L, Nordström P, Zidén L, Stenström K (2018). Treatment of humerus fractures in the elderly: A systematic review covering effectiveness, safety, economic aspects and evolution of practice. PLoS One.

[65] Guyatt GH, Oxman AD, Kunz R, Vist GE, Falck-Ytter Y, Schunemann HJ (2008). What is "quality of evidence" and why is it important to clinicians?. BMJ.

[66] Napier C, McCormack RG, Hunt MA, Brooks-Hill A (2013). A physiotherapy triage service for orthopaedic surgery: an effective strategy for reducing wait times. Physiother Can.

[67] Marks D, Comans T, Thomas M, Ng SK, O'Leary S, Conaghan PG, Scuffham PA, Bisset L (2016). Agreement between a physiotherapist and an orthopaedic surgeon regarding management and prescription of corticosteroid injection for patients with shoulder pain. Man Ther.

[68] Jovic D, Mulford J, Ogden K, Zalucki N (2019). Diagnosis and management of chronic hip and knee pain in a Tasmanian orthopaedic clinic: a study assessing the diagnostic and treatment planning decisions of an advanced scope physiotherapist. Aust J Prim Health.

[69] Lowry V, Bass A, Lavigne P, Léger-St-Jean B, Blanchette D, Perreault K, Roy JS, Aiken A, Décary S, Desmeules F (2020). Physiotherapists' ability to diagnose and manage shoulder disorders in an outpatient orthopedic clinic: results from a concordance study. J Shoulder Elb Surg.

[70] Dickens V, Ali F, Gent H, Rees A (2003). Assessment and diagnosis of knee injuries: the value of an experienced physiotherapist. Physiotherapy.

[71] MacKay C, Davis AM, Mahomed N, Badley EM (2009). Expanding roles in orthopaedic care: a comparison of physiotherapists and orthopaedic surgeon recommendations for triage. J Eval Clin Pract.

[72] Razmjou H, Robarts S, Kennedy D, McKnight C, MacLeod AM, Holtby R (2013). Evaluation of an advanced-practice physical therapist in a specialty shoulder clinic: diagnostic agreement and effect on wait times. Physiother Can.

[73] Ashmore K, Smart K, O'Toole G, Doody C (2014). Triage of knee pain by an extended scope physiotherapist (ESP) in an orthopaedic clinic: a clinical audit. Physiother Pract Res.

[74] Twizeyemariya A, Morris JH, Grimmer K (2018). What is the current evidence for cost of waiting on the outpatient list for management/treatment of orthopaedic/musculoskeletal complaints? A systematic review. Rheumatol Orthop Med.

[75] Morris J, Twizeyemariya A, Grimmer K (2018). What is the current evidence of the impact on quality of life whilst waiting for management/treatment of orthopaedic/musculoskeletal complaints? A systematic scoping review. Qual Life Res.

[76] Fennelly O, Blake C, FitzGerald O, Caffrey A, Fletcher L, Smart K, Corcoran S, Shé ÉN, Casserley-Feeney S, Desmeules F, et al. Advanced musculoskeletal physiotherapy practice: The patient journey and experience. Musculoskeletal Science and Practice. 2020;45. 10.1016/j.msksp.2019.102077 31731056

[77] Fennelly O, Blake C, FitzGerald O, Breen R, O'Sullivan C, O'Mir M, Desmeules F, Cunningham C (2018). Advanced musculoskeletal physiotherapy practice in Ireland: a National Survey. Musculoskeletal Care.

[78] O'Mahony N, Blake C (2017). Musculoskeletal triage: the experiences of advanced practice physiotherapists in Ireland. Physiotherapy Practice and Research.

[79] Fennelly O, Desmeules F, O'Sullivan C, Heneghan NR, Cunningham C (2020). Advanced musculoskeletal physiotherapy practice: Informing education curricula. Musculoskeletal Science and Practice.

[80] Fennelly O, Blake C, Desmeules F, Stokes D, Cunningham C (2018). Patient-reported outcome measures in advanced musculoskeletal physiotherapy practice: a systematic review. Musculoskeletal Care.

