# Supplementary material for: Effects on health and process outcomes of physiotherapist-led orthopaedic triage for patients with musculoskeletal disorders: a systematic review of comparative studies
Source: BMC Musculoskelet Disord. 2020 Oct 10;21:673. doi: 10.1186/s12891-020-03673-9 (PMC7548042; doi:10.1186/s12891-020-03673-9)
Supplement: Supplementary file 1 — Additional file 1. Modified Downs and Black checklist. [file 12891_2020_3673_MOESM1_ESM.docx]

# Additional file 1. Modified Downs & Black Checklist

Item 27 (study power) has been modified; it was rated for whether a power calculation was performed, with a maximum score of 28 (instead of 32) for RCTs and 25 for non-randomised studies.

| **Number** | **Criteria** | **Scoring Criteria** | **Score** |
| --- | --- | --- | --- |
| 1 | Is the hypothesis/aim/objective of the study clearly described? | A point was given if the hypothesis, aim or objective of the study was implicitly or explicitly indicated anywhere in the article. | 0=No 1=Yes |
| 2 | Are the main outcomes to be measured clearly described in the "Introduction" or "Methods" section? | A point was given if the main outcomes to be measured were clearly described in the "Introduction" or "Methods" section. | 0=No 1=Yes |
| 3 | Are the characteristics of the patients included in the study clearly described? | A point was given if the inclusion or exclusion criteria, or both, were indicated. | 0=No 1=Yes |
| 4 | Are the interventions of interest clearly described? | A point was given if the criteria for PT-led orthopaedic triage were described in detail. | 0=No 1=Yes |
| 5 | Are the distributions of principal confounders for each group of participants to be compared clearly described? | Two points were awarded if a study reported any possible confounders (e.g., sex ratios, age, comorbidities, and severity of injury) that might account for differences between groups clearly in table format. One point was awarded if the study indicated that groups were matched for any such demographical variables or if potential confounders were mentioned in the text of the article but not clearly listed in table format. No points were awarded if the study did not report any confounders. | 0=No 1=Partially 2=Yes |
| 6 | Are the main findings of the study clearly described? | A point was awarded if quantitative data were reported for all of the main outcome measures. | 0=No 1=Yes |
| 7 | Does the study provide estimates of the random variability in the data for the main outcomes? | A point was awarded if the interquartile range (for non-normally distributed data), standard error, standard deviation, or confidence intervals (for normally distributed data) were reported. If the distribution of the data was not described, we assumed that the estimates used were appropriate, and we answered "yes" (1 point). | 0=No 1=Yes |
| 8 | Have all of the important adverse events that may be a consequence of the intervention been reported? | A point was awarded if any adverse events were explicitly indicated | 0=No 1=Yes |
| 9 | Have the characteristics of patients lost to follow-up been describe? | A point was awarded if a study explicitly reported the number and reason for patients lost to follow-up or where losses to follow-up were so small that findings would be unaffected by their inclusion. A point was also awarded if patients were included in the study were assessed and evaluated as planned. | 0=No 1=Yes |
| 10 | Have actual probability values been reported (e.g. 0.035 rather than < 0.05) for the main outcomes except where the probability value is less than 0.001? | A point was awarded if the exact P value was provided for both statistically significant and non-significant results for at least the main outcome measures. | 0=No 1=Yes |
| **EXTERNAL VALIDITY** | | | |
| 11 | Were the subjects asked to participate in the study representative of the entire population from which they were recruited? | A point was awarded if the study identified the source population for patients and described how the patients were selected. Patients were determined to be representative if they comprised the entire source population, an unselected sample of consecutive patients, or a random sample (only feasible where a list of all members of the relevant population exists). | 1=Yes 0=No 0=Unable to determine |
| 12 | Were those subjects who were prepared to participate representative of the entire population from which they were recruited? | A point was awarded if the proportion of patients included in the study were representative of the population and number or proportion of those asked who agreed to participate was stated. | 1=Yes 0=No 0=Unable to determine |
| 13 | Were the staff, places and facilities where the patients were treated, representative of the treatment the majority of patients receive? | A point was awarded if the study demonstrated that the intervention was representative of that in use in the source population; i.e. not specialist centres unrepresentative of the hospitals most of the source population would attend. | 1=Yes 0=No 0=Unable to determine |
| **INTERNAL VALIDITY - BIAS** | | | |
| 14 | Was an attempt made to blind study subjects to the intervention they have received? | A point was awarded if the study specifically stated that the patients were unaware of which intervention they received. | 1=Yes 0=No 0=Unable to determine |
| 15 | Was an attempt made to blind those measuring the main outcomes of the intervention? | A point was awarded if the study specifically stated that those assessing the outcome measures were unaware of (or would have no way of knowing) assigned groups. | 1=Yes 0=No 0=Unable to determine |
| 16 | If any of the results of the study were based on "data dredging", was this made clear? | A point was awarded if no retrospective unplanned (at the outset of the study) subgroup analyses were reported. | 1=Yes 0=No 0=Unable to determine |
| 17 | In trials and cohort studies, do the analyses adjust for different lengths of follow-up of patients, or in case-control studies, is the time period between the intervention and outcome the same for cases and controls? | A point was awarded if the follow-up was the same for all study patients, or, for agreement-studies, if all patients was assessed by both care-givers at approximately the same time i.e. within a couple of weeks. If different lengths of follow-up were adjusted for, one point was awarded. | 1=Yes 0=No 0=Unable to determine |
| 18 | Were the statistical tests used to assess the main outcomes appropriate? | A point was awarded if the distribution of the data (normal or not) was described. If the distribution of the data was not described, we assumed that the estimates used were appropriate and the question was answered “yes”. | 1=Yes 0=No 0=Unable to determine |
| 19 | Was compliance with the intervention/s reliable? | A point was awarded where there was compliance with the allocated treatment and no contamination between groups. | 1=Yes 0=No 0=Unable to determine |
| 20 | Were the main outcome measures used accurate (valid and reliable)? | A point was awarded if the primary outcome measures were thought to be valid and reliable. | 1=Yes 0=No 0=Unable to determine |
| **INTERNAL VALIDITY - CONFOUNDING (SELECTION BIAS)** | | | |
| 21 | Were the patients in different intervention groups (trials and cohort studies) recruited from the same population? | A point was awarded when participants from all comparison groups were recruited from the same population. | 1=Yes 0=No 0=Unable to determine |
| 22 | Were study subjects in different intervention groups (trials and cohort studies) recruited over the same period of time? | A point was awarded when the study provided a specific time line for patient recruitment (prospective studies). | 1=Yes 0=No 0=Unable to determine |
| 23 | Were study subjects randomized to intervention groups? | A point was awarded if random allocation of patients was stated in the “Method” section of the article. | 1=Yes 0=No 0=Unable to determine |
| 24 | Was the randomized intervention assignment concealed from both patients and healthcare staff until recruitment was complete and irrevocable? | A point was awarded if the study was randomised and assignment was concealed from patients and staff. | 1=Yes 0=No 0=Unable to determine |
| 25 | Was there adequate adjustment for confounding in the analyses from which the main findings were drawn? | A point was awarded unless the effect of the main confounders was not investigated or confounding was demonstrated, but no adjustment was made in the final analyses. | 1=Yes 0=No 0=Unable to determine |
| 26 | Were losses of patients to follow-up taken into account? | A point was awarded if the numbers of patients lost to follow-up were reported or if the proportion lost to follow-up was too small to affect the main findings. | 1=Yes 0=No 0=Unable to determine |
| **POWER** | | | |
| 27 | Was a power calculation performed? | A point was awarded if a power calculation was performed prior to inclusion of patients. | 1=Yes 0=No |
|  |  | **Overall Max Possible Score** | 28 |
