# Supplementary material for: Effects on health and process outcomes of physiotherapist-led orthopaedic triage for patients with musculoskeletal disorders: a systematic review of comparative studies
Source: BMC Musculoskelet Disord. 2020 Oct 10;21:673. doi: 10.1186/s12891-020-03673-9 (PMC7548042; doi:10.1186/s12891-020-03673-9)
Supplement: Supplementary file 2 — Additional file 2. Excluded publications. [file 12891_2020_3673_MOESM2_ESM.docx]

**Additional file 2 – Excluded publications**

Publications excluded for the purpose of the analysis of Physiotherapist-led orthopaedic triage.

*Excluded publications due to high risk of bias*

Aiken, A. B., et al. (2008). "Easing the burden for joint replacement wait times: the role of the expanded practice physiotherapist." Healthcare quarterly (Toronto, Ont.) 11(2): 62-66.

Burn, D. and E. Beeson (2014). "Orthopaedic triage: Cost effectiveness, diagnostic/surgical and management rates." Clinical Governance 19(2): 126-136.

Kerridge-Weeks, M. and N. J. Langridge (2016). "Orthopaedic spinal triage: An evaluation of decision making and outcome in a physiotherapy led service." International Journal of Health Governance 21(1): 5-17.

*Excluded publications due to study design*

Bath, B., et al. (2012). "A spinal triage programme delivered by physiotherapists in collaboration with orthopaedic surgeons." Physiotherapy Canada 64(4): 356-366.

Blackburn, M. S., et al. (2009). "Physiotherapy-led triage clinic for low back pain." Australian Health Review 33(4): 663-670.

Caffrey, A., et al. (2019). "Physiotherapist-Led Triage at a Rheumatology-Based Musculoskeletal Assessment Clinic: an 18-Month Service Evaluation of Activity and Outcomes." ACR open rheumatology 1(4): 213-218.

Comans, T., et al. (2014). "Cost-effectiveness of a physiotherapist-led service for orthopaedic outpatients." Journal of Health Services Research and Policy 19(4): 216-223.

Gardiner, J. and P. Turner (2002). "Accuracy of clinical diagnosis of internal derangement of the knee by extended scope physiotherapists and orthopaedic doctors: Retrospective audit." Physiotherapy 88(3): 153-157.

’Farrell, S., et al. (2013). "Orthopaedic triage at a physiotherapist-led ‘Musculoskeletal Assessment Clinic’: A seven-month service evaluation of outcomes." Irish Journal of Medical Science 183(4): 565-571.

Oakes, H. (2009). "Orthopaedic shoulder clinic diagnosis and treatment plan audit." Clinical Governance 14(2): 126-133.

Parfitt, N., et al. (2012). "Direct listing for total hip replacement (THR) by primary care physiotherapists." Clinical Governance 17(3): 210-216.

Parmar, V., et al. (2015). "Comparison of referrals for lumbar spine magnetic resonance imaging from physiotherapists, primary care and secondary care: How should referral pathways be optimised?" Physiotherapy (United Kingdom) 101(1): 82-87.

Pearse, E. O., et al. (2006). "The extended scope physiotherapist in orthopaedic out-patients - An audit." Annals of the Royal College of Surgeons of England 88(7): 653-655.

Rabey, M., et al. (2009). "Orthopaedic physiotherapy practitioners: Surgical and radiological referral rates." Clinical Governance: An International Journal 14(1): 15-19.

Ronan, L. and J. R. E. Ramsay (2014). "A comparison of diagnostic accuracy in the identification of meniscal tears between extended scope practitioners and consultant orthopaedic surgeons." Physiotherapy Practice and Research 36(1): 55-64.

Smyth, C., et al. (2019). "Physiotherapist-led triage of patients with thoracic spine pain in a musculoskeletal assessment clinic: A service evaluation of activity and outcomes." Physiotherapy Practice and Research 40(2): 145-153.

Wood, L., et al. (2016). "A review of the surgical conversion rate and independent management of spinal extended scope practitioners in a secondary care setting." Annals of the Royal College of Surgeons of England 98(3): 187-191.

*Excluded publications due to Population*

Décary, S., et al. (2017). "Diagnostic validity and triage concordance of a physiotherapist compared to physicians' diagnoses for common knee disorders." BMC Musculoskeletal Disorders 18(1).

*Excluded publications due to Intervention*

Cuschieri, S., et al. (2014). "Outcome of low back pain patients referred to orthopeadic outpatient clinic." Malta Medical Journal 26(2): 52-57.

Marks, D., et al. (2016). "Increasing Capacity for the Treatment of Common Musculoskeletal Problems: A Non-Inferiority RCT and Economic Analysis of Corticosteroid Injection for Shoulder Pain Comparing a Physiotherapist and Orthopaedic Surgeon." PloS One 11(9): e0162679.

Patel, S., et al. (2011). "The accuracy of primary care teams in diagnosing disorders of the shoulder." Journal of Evaluation in Clinical Practice 17(1): 118-122.

Rymaszewski, L. A., et al. (2005). "A team approach to musculo-skeletal disorders." Annals of the Royal College of Surgeons of England 87(3): 174-180.

Voorn, V. M. A., et al. (2013). "An innovative care model coordinated by a physical therapist and nurse practitioner for osteoarthritis of the hip and knee in specialist care: A prospective study." Rheumatology International 33(7): 1821-1828.

Yin, D., et al. (2019). "Can a physiotherapy student assume the role of an advanced practice physiotherapist in Orthopaedic surgery triage? A prospective observational study." BMC Musculoskeletal Disorders 20(1): 498.

*Excluded publications due to no Comparison*

Aiken, A. B., et al. (2009). "Role of the advanced practice physiotherapist in decreasing surgical wait times." Healthcare quarterly (Toronto, Ont.) 12(3): 80-83.

MacKay, C., et al. (2012). "Physical therapists working in expanded roles in orthopaedic clinics: Impact on non-surgical patients with arthritis." Osteoarthritis and Cartilage 20: S166.

Murphy, S., et al. (2013). "The role of clinical specialist Physiotherapists in the management of low back pain in a Spinal Triage Clinic." Irish journal of medical science: 1-8.

*Excluded publications due to format (poster or no full text available)*

Allan, P., et al. (2017). "Impact of an advanced physiotherapy practitioner-led pilot community spinal MSK service." Physiotherapy (United Kingdom) 103: e74-e75.

rennan, A., et al. (2016). "The development of physiotherapy-led musculoskeletal triage services in Ireland." Annals of the rheumatic diseases 75: 1275.

Byles, S. and R. Ling (1989). "Orthopaedic Out-patients — A Fresh Approach." Physiotherapy (United Kingdom) 75(7): 435-437.

Chan, A., et al. (2017). "Integrated pain assessment and spinal service in west berkshire successfully delivers improved outcomes for musculoskeletal pain." Rheumatology (United Kingdom) 56: ii171.

Dunstan, E. and L. Wood (2016). "The utility of esp triage in a specialist secondary care spinal centre: A service evaluation." Manual Therapy 25: e115-e116.

Fennelly, O., et al. (2017). "HPR advanced practice musculoskeletal physiotherapy services: A national evaluation." Annals of the rheumatic diseases 76: 1493.

Grimmer-Somers, K., et al. (2012). "Multidisciplinary musculoskeletal triage clinic: Outcomes 12 months on." Internal medicine journal 42: 36.

Harrison, J., et al. (2001). "Reducing waiting times: Physiotherapy shoulder assessment clinic." International Journal of Therapy and Rehabilitation 8(2): 57-59.

Hattam, P. and A. Smeatham (1999). "Evaluation of an orthopaedic screening service in primary care." Clinical Performance and Quality Health Care 7(3): 121-124.

Hensman-Crook, A. (2017). "Advanced physiotherapy in primary care. Part of the solution for a growing crisis?" Physiotherapy (United Kingdom) 103: e112.

Higginson, R. and C. Hutchinson (2017). "Diagnostic accuracy of knee pathologies by a telephone based Advanced Level Physiotherapy service." Physiotherapy (United Kingdom) 103: e58.

Hockin, J. and G. Bannister (1994). "The Extended Role of a Physiotherapist in an Out-patient Orthopaedic Clinic." Physiotherapy (United Kingdom) 80(5): 281-284.

Hy, M. J., et al. (2016). "Back pain assessment clinic based in primary care: A'safe, effective and cost-saving model. Results of a'12-month pilot project." Internal medicine journal 46: 6.

Lewis, A., et al. (2010). "Survey of MRI requests from an acute spinal assessment service." Rheumatology 49: i128-i129.

McNeilly, N. E. and J. Waterfield (2012). "A service evaluation of a physiotherapy extended scope practitioner community-led injection service." Rheumatology (United Kingdom) 51: iii59.

Ryan, L., et al. (2015). "The benefits of an MSK clinical specialist physiotherapy (CSP) led triage service in reducing an orthopaedic/rheumatology waiting list in University Hospitals of Limerick (UHL)." Irish journal of medical science 184: S207-S208.

Samsson, K. S. and M. E. H. Larsson (2015). "Good perceived quality of physiotherapy triage assessment of patients referred for orthopaedic consultation." Physiotherapy (United Kingdom) 101: eS1331-eS1332.

Seth, B. and A. Natarajan (2014). "Integrated pathway for management of spinal pain in the national health service (NHS) - The uk experience." Pain Practice 14: 44.

Smith, D. and M. Raymer (2011). "From implementation to integration-physiotherapist-led model changes the face of orthopaedics in Australia: The journey from Vancouver to Amsterdam." Physiotherapy (United Kingdom) 97: eS1597-eS1598.

Smith, J., et al. (2016). "Clinical and cost-effectiveness of a physiotherapy led case management service for back pain." Physiotherapy 102: e133-e134.

Soever, L., et al. (2011). "Evaluation results utilizing advanced practice physiotherapist in rural Canadian family practice for patients with hip and knee arthritis." Physiotherapy (United Kingdom) 97: eS1601.

Weale, A. E. and G. C. Bannister (1995). "Who should see orthopaedic outpatients--physiotherapists or surgeons?" Annals of the Royal College of Surgeons of England 77(2 Suppl): 71-73.
